# Genomic characterisation of the overlap of endometriosis with 76 comorbidities identifies pleiotropic and causal mechanisms underlying disease risk

**DOI:** 10.1007/s00439-023-02582-w

**Published:** 2023-07-06

**Authors:** Isabelle M. McGrath, International Endometriosis Genetics Consortium, Grant W. Montgomery, Sally Mortlock

**Affiliations:** The Institute for Molecular Bioscience, The University of Queensland, Brisbane, QLD 4072 Australia

## Abstract

Comorbid conditions can be driven by underlying pleiotropic and causal mechanisms that can provide insights into shared molecular and biological processes contributing to disease risk. Endometriosis is a chronic condition affecting one in nine women of reproductive age and poses many challenges including lengthy diagnostic delays and limited treatment efficacy owing to poor understanding of disease aetiology. To shed light on the underlying biological mechanisms and to identify potential risk factors, we examine the epidemiological and genomic relationship between endometriosis and its comorbidities. In the UK Biobank 292 ICD10 codes were epidemiologically correlated with endometriosis diagnosis, including gynaecological, immune, infection, pain, psychiatric, cancer, gastrointestinal, urinary, bone and cardiovascular traits. A subset of the identified comorbidities (*n* = 76) underwent follow-up genetic analysis. Whilst Mendelian randomisation suggested causality was not responsible for most comorbid relationships, 22 traits were genetically correlated with endometriosis, including pain, gynaecological and gastrointestinal traits, suggestive of a shared genetic background. Pleiotropic genetic variants and genes were identified using gene-based and colocalisation analysis. Shared genetic risk factors and potential target genes suggest a diverse collection of biological systems are involved in these comorbid relationships including coagulation factors, development of the female reproductive tract and cell proliferation. These findings highlight the diversity of traits with epidemiological and genomic overlap with endometriosis and implicate a key role for pleiotropy in the comorbid relationships.

**Supplementary Information:**

The online version contains supplementary material available at 10.1007/s00439-023-02582-w.

## Introduction

Endometriosis is a common condition affecting 1 in 9 women of reproductive age (Rowlands et al. 2021). Characterised by the presence of endometrial-like tissue outside of the endometrium, it is associated with chronic pelvic pain and infertility. Historically endometriosis was considered a gynaecological disease, although is now understood to be a chronic systemic disease (Taylor et al. 2021). Studies have estimated that 50% of the variation in endometriosis risk is captured by genetics and the remaining 50% is influenced by environmental and lifestyle factors (Saha et al. 2015; Treloar et al. 1999). Genome-wide association studies (GWASs) have identified 42 regions of the genome associated with endometriosis risk however the biological mechanisms contributing to the development of endometriosis remain poorly understood (Rahmioglu et al. 2023). There are few known risk factors for endometriosis and there is substantial variation in the severity of symptoms between individuals. Definitive diagnosis requires surgical visualisation however, the diagnostic surgery fails to identify endometriosis in one in four instances (Fernando et al. 2013; Kazanegra et al. 2008; Stegmann et al. 2008). Additionally, endometriosis often presents alongside multiple other conditions (Blass et al. 2022; Choi et al. 2017; Lee et al. 2019; Parazzini et al. 2017; Peters et al. 2022). For many of these conditions, it is unknown whether there is a biological basis for their comorbidity.

The coexistence of two or more conditions can suggest common aetiological pathways and therefore investigation into comorbid relationships can provide novel insights into underlying molecular disease mechanisms and biology (Ko et al. 2016). Large-scale epidemiological studies have implicated many traits as comorbid with endometriosis, including menstrual characteristics, infertility, gastrointestinal diseases, gynaecological malignancies, uterine fibroids and immunological diseases (Blass et al. 2022; Choi et al. 2017; Lee et al. 2019; Parazzini et al. 2017; Peters et al. 2022). The putative shared underlying biology has been investigated for a subset of these traits using genomic datasets (Adewuyi et al. 2020, 2021, 2022, 2023; Gallagher et al. 2019; Garitazelaia et al. 2021; Kho et al. 2021; Koller et al. 2023; Masuda et al. 2020; Mills et al. 2021; Mortlock et al. 2022; Painter et al. 2011; Rahmioglu et al. 2015; Rueda-Martinez et al. 2021; Venkatesh et al. 2022; Yan et al. 2022; Yang et al. 2021; Yarmolinsky et al. 2019). In addition to using the overlap between endometriosis and other traits to understand the disease aetiology, it can also be used to inform clinical predictors and outcomes of disease.

The availability of large-scale health and genetic information from biobanks provides a unique opportunity to investigate relationships between comorbid conditions in the population. In this study, we performed a search for comorbidities of endometriosis in the UK Biobank (UKB) and investigated the comorbid nature of these conditions using genomic approaches including genetic correlation, Mendelian randomisation (MR), colocalisation analysis and gene-based analysis.

## Methods

### UK Biobank comorbidity search

A search for epidemiological comorbidities of endometriosis was conducted in the UKB, a large population database containing phenotypic and genotypic information for half a million individuals aged between 40–69 years when recruited between 2006 and 2010 in the United Kingdom. The UKB cohort was restricted to unrelated European females (defined using genetic information; Genetic Relationship Matrix < 0.05), from which two endometriosis cohorts were defined. The first cohort included endometriosis cases with entries in 132122-0.0 and 132123-0.0 (Date and Source of N80 first report; includes both surgically diagnosed and self-report). The second cohort included endometriosis cases with an ICD10 diagnostic code, likely to represent a surgical diagnosis. From both cohorts, individuals with the ICD10 diagnostic code N80.0: endometriosis of the uterus, and no other endometriosis diagnosis (N80.1-N80.9) were excluded. N80.0 refers to adenomyosis, which is currently recognised as a distinct disease to endometriosis. Controls (unrelated females without any N80.x diagnosis) were matched to cases by year of birth (field 34-0.0), whereby individuals born between 1937 and 1970 were included. Chi-square test and Fisher’s exact test, where appropriate, were used to compare the frequency of non-N80 ICD10 diagnoses between cases (*n* = 5432) and controls (*n* = 92,344) for the first cohort, and cases (*n* = 2085) and controls (*n* = 52,125) for the second cohort was performed. Significant ICD10 codes were declared at false discovery rate (FDR) of 5%. The age of diagnosis for endometriosis was calculated using the Date of N80 first report (132122-0.0) and the Month (52-0.0) and Year of Birth (34-0.0) columns. The date of birth was approximated as the 15th of the month. The diagnosis date of the comorbidities was calculated in the same manner using the corresponding ICD10 diagnosis date.

An additional analysis utilising the endometriosis ICD9 and ICD10 subcode information was performed. The ICD10 codes were compared using chi-square test between individuals with Endometriosis of ovary (*n* = 679) and individuals with Endometriosis of pelvic peritoneum (*n* = 502). Individuals with both Endometriosis of ovary and Endometriosis of pelvic peritoneum were excluded from the analysis. Other endometriosis subcodes were not considered due to limited power/not of interest.

### GWAS summary statistics attainment

Summary statistics from seven European cohorts included in the Sapkota et al. 2017 meta-analysis (14,926 cases; 189,715 controls) of endometriosis were meta-analysed alongside endometriosis GWAS summary statistics obtained from FinnGen Release 5 (Phenotype: “Endometriosis”, 8288 cases; 68,969 controls) (Kurki et al. 2022). The meta-analysis was performed using inverse variance-weighted fixed-effect model in METAL (Willer et al. 2010). SNPs were filtered to only include those with a consistent direction of effect in at least three cohorts. A genome-wide significance threshold of *P* < 5 × 10^−8^ was used to define significantly associated variants and an *R*^2^ of > 0.6 was used to define independent loci (Watanabe et al. 2017).

Genome-Wide Association Study (GWAS) summary statistics were attained for 76 traits (Supplementary Table 1). These traits were selected through association with endometriosis in the epidemiological comorbidity search (Supplementary Tables 2, 3) whereby data generated from at least 1000 cases of European ancestry were available. GWA summary statistics for all traits were cleaned to ensure missing SNP sample size was replaced with the published sample size, rsIDs were annotated, and SNPs with imputation quality > 0.9 were retained. In the case of duplicated rsIDs, the variant with the lowest p-value was retained.

### Genetic correlation analyses

Genetic correlation was performed using LDSC (v1.0.1) and precomputed linkage disequilibrium (LD) scores from the 1000 Genomes European reference set (https://data.broadinstitute.org/alkesgroup/LDSCORE/eur_w_ld_chr.tar.bz2). The alleles in the GWAS summary statistics for each trait were crosschecked against HapMap3 SNPs (https://data.broadinstitute.org/alkesgroup/LDSCORE/w_hm3.snplist.bz2) used to estimate the LD scores using the --merge-alleles function. Genetic correlation was assessed for 74 of 76 traits, as the mean chi-squares were too small for two traits (dyspepsia and cystitis). Significant results are declared at a FDR of 5%.

### GWAS-PW

Colocalisation analysis was performed with GWAS-PW v0.21, which performs a genome-wide scan for genomic regions with evidence of a shared causal variant between traits (Pickrell et al. 2016). Specifically, it evaluates the probability of four hypotheses: the region contains a genetic variant associated only with Trait 1 (PPA1), associated only with Trait 2 (PPA2), associated with both Trait 1 and Trait 2 (PPA3), or contains two genetic variants independently associated with Trait 1 and Trait 2 (PPA4) (Pickrell et al. 2016). GWAS-PW analysis was performed for endometriosis against the 22 traits identified to have a significant genetic correlation with endometriosis. The summary statistics for each comorbidity were merged with the endometriosis summary statistics by the rsID and alleles. Test statistics were harmonised so that the association statistics referred to the same allele. *Z* scores (beta/standard error (SE)) and variances (SE^2^) for each SNP were provided as input to GWAS-PW. The presence of an endometriosis risk variant within regions identified by GWAS-PW was achieved by analysing the endometriosis GWAS summary statistics with the SNP2GENE function in FUMA (Watanabe et al. 2017).

### fastBAT

Gene-based analysis was performed using fastBAT analysis as implemented in gcta_1.93.3beta2 for the 22 traits with a significant genetic correlation with endometriosis. fastBAT performs a set-based association analysis, identifying genes enriched for nearby risk variants. QIMRHCS (Sapkota et al. 2017) was used as the LD reference panel. SNPs with minor allele frequency > 0.01 were retained for analysis. Analysis was run against the recommended gene list (https://yanglab.westlake.edu.cn/software/gcta/res/glist-hg19.txt). Genes significantly associated with each trait (*P* < 0.05/n genes), as well as the top 1% of genes associated with each trait were compared across traits. Genes were identified by their name and genomic location in cross-comparisons.

### Mendelian randomisation

MR, as implemented in MR-Base (TwoSampleMR version 0.5.6), was performed using the MR Egger, inverse-variance weighted (IVW) and weighted median methods with endometriosis both as the exposure and outcome variable. The IVW method assumes all variants are valid instrumental variables, whereas MR-Egger relaxes this assumption to allow for an overall directional pleiotropic effect, which is able to provide valid estimates with the condition that the pleiotropic effects on the outcome is independent of their effects on the exposure (Burgess et al. 2013). Weighted median method provides valid estimates if at least 50% of the variants are valid instrumental variables (Bowden et al. 2016). The MR-Egger and weighted median methods are important sensitivity tests for MR analysis. Studies which reported odds ratio (OR) and 95% confidence interval (CI) were converted to beta and SE via beta = log(OR), SE = (log(CI Upper))−log(CI Lower)/3.92 for MR analysis. Significant SNP associations with the exposure at the genome-wide significance threshold (*P* < 5 × 10^−8^) were selected, data was LD clumped using the 1000 Genomes European reference data (*r*^2^ < 0.001, window 10,000 kb), then the exposure data was harmonised with the outcome data. Analyses were run for exposures with at least three significant SNPs. The intercept was calculated to test overall directional pleiotropy via the MR Egger method. To test whether individual SNPs showed heterogeneity, Cochran’s *Q* and Rücker’s *Q*′ statistics were calculated for the IVW and MR-Egger methods, respectively. The strength of the SNPs selected as instrumental variables were evaluated by F-statistics, calculated by finding the mean of beta^2^/SE^2^ across all IVs for each exposure.

A bidirectional MR was run with a second tool: Generalised Summary-data-based Mendelian Randomisation (GSMR) implemented in the GCTA (v1.93.3beta2) software (Zhu et al. 2018). Due to the absence of allele frequency in the summary statistics for nine traits, GSMR analysis with endometriosis was not performed for all traits. Analyses were run for traits with at least three significant (*P* < 5 × 10^−8^) associations with all other default settings. Individual-level genetic data for 5185 European individuals from the QIMRHCS cohort (Sapkota et al. 2017) was used as the LD reference sample. GSMR has an in-built feature, HEIDI-outlier, for detection and removal of pleiotropic SNPs. Even if pleiotropic SNPs with small effects remain, the estimate is not biased by pleiotropy (Zhu et al. 2018). GSMR is also unbiased by sample overlap between the exposure and the outcome variable (Revez et al. 2020).

*P*-values were declared significant at stringent thresholds of *P* < 6.58 × 10^−4^ (0.05/76) with endometriosis as the exposure, and *P* < 1.22 × 10^−3^ (0.05/41) with endometriosis as the outcome. For significant causal relationships identified by IVW, MR-Egger, weighted median or GSMR, additional sensitivity testing was conducted to further assess the robustness. MR-PRESSO global, outlier and distortion tests were performed as an additional sensitivity test using run_mr_presso from the TwoSampleMR package. MR-PRESSO can identify variants showing evidence of horizontal pleiotropy, correct for these variants by removing them, and compare the difference in the causal estimates with and without these variants (Verbanck et al. 2018). Default parameters were applied, except for waist-to-hip ratio (WHR) and waist-to-hip ratio adjusted for BMI (WHRadjBMI) as the exposure variable, for the NbDistribution was 5000, as 1000 was insufficient. The MR Steiger method was also conducted using the command steiger_filtering from the TwoSampleMR package. This method tests whether the SNP-outcome association is greater than the SNP-exposure association, as this may be evidence for a direct effect of the SNP on the outcome variable (Hemani et al. 2017). Leave-one-out sensitivity testing was conducted when IVW, MR-Egger, or weighted median method were significant. Leave-one-out sensitivity testing recalculates the causal estimate upon individual removal of each SNP to determine whether the relationship may be being driven by an individual SNP. Where appropriate, causal tests were repeated with the SNPs passing leave one out analysis and MR-Steiger testing.

## Results

### Endometriosis comorbidities identified in the UKB

The frequency of ICD10 codes were compared between endometriosis cases (*n* = 5432) and controls (*n* = 92,344) in the UKB. A total of 292 ICD10 codes were declared significant at an FDR of 5% (Supplementary Table 2). Most of these codes occurred at greater frequency in the case cohort to the control cohort: only five codes related to pregnancy were more prevalent in the control cohort. Of the 21 ICD10 code chapters, 16 were represented across the 292 significant ICD10 codes. The most common chapter was Chapter 14: Diseases of the genitourinary system (*n* = 59 ICD10 codes), followed by Chapter 21: Factors influencing health status and contact with health services (*n* = 53), Chapter 13: Diseases of the musculoskeletal system and connective tissue (*n* = 41), Chapter 11: Diseases of the digestive system (*n* = 40), and Chapter 18: Symptoms, signs and abnormal clinical and laboratory findings, not elsewhere classified (*n* = 36). The top five traits were N73.6 Female pelvic peritoneal adhesions (11.03% of cases, 0.95% of controls), N83.2 Other and unspecified ovarian cysts (9.68% of cases, 1.67% of controls), D25.9 Leiomyoma of uterus, unspecified (11.84% of cases, 3.48% of controls), N94.6 Dysmenorrhoea, unspecified (4.27% of cases, 0.65% of controls), and Z90.7 Acquired absence of genital organ(s) (8.04% of cases, 2.07% of controls). The average age of endometriosis diagnosis was 39.2 ± 9.36 years (mean ± SD), which increased to 44.9 ± 7.70 years when only cases with an ICD10 diagnosis were considered. Of the 292 significant ICD10 codes, there are 10 ICD10 codes with a mean age of diagnosis in endometriosis cases before the mean age of endometriosis diagnosis: O47.9 False labour, unspecified, O80.0 Spontaneous vertex delivery, O70.1 Second degree perineal laceration during delivery, Z31.2 In vitro fertilisation, O41.8 Other specified disorders of amniotic fluid and membranes, Z37.0 Single live birth, O70.0 First degree perineal laceration during delivery, N97.9 Female infertility, unspecified, N94.5 Secondary dysmenorrhoea, and Z31.4 Procreative investigation and testing. The comorbid conditions are generally diagnosed earlier in endometriosis cases than endometriosis controls (*P* = 4.7 × 10^−5^). When the case cohort was restricted to cases with an ICD10 endometriosis diagnosis (2085 cases, 52,125 controls), 281 ICD10 codes were significant (FDR 5%) (Supplementary Table 3). Seventy ICD10 codes only reached significance in this restricted endometriosis analysis.

To ascertain whether different endometriosis lesion sites are associated with different comorbidities, individuals with endometriosis of the ovary were compared to individuals with endometriosis of the pelvic peritoneum. The groups were exclusive of one another. To maximise power utilising smaller cohorts, ICD10 codes were simplified to their base codes. Seven codes significantly differed in frequency (FDR 5%) between individuals with endometriosis of the ovary and endometriosis of the pelvic peritoneum (Table 1). “N94 Pain and other conditions associated with female genital organs and menstrual cycle”, “N97 Female infertility” and “Z30 Contraceptive management” were more frequent in individuals with endometriosis of the pelvic peritoneum than endometriosis of the ovary. “D25 Leiomyoma of uterus”, “N83 Noninflammatory disorders of ovary, fallopian tube and broad ligament”, “N70 Salpingitis and oophoritis” and “Z09 Encounter for follow-up examination after completed treatment for conditions other than malignant neoplasm” were more frequent in individuals with endometriosis of the ovary than endometriosis of the pelvic peritoneum.

**Table 1:** ICD10 codes with different frequencies between individuals with endometriosis of the ovary (*n* = 679) and endometriosis of the pelvic peritoneum (*n* = 502) in the UKB

ICD10 base code	Frequency in endometriosis of the pelvic peritoneum (%)	Frequency in endometriosis of the ovary (%)	*P* value
D25: Leiomyoma of uterus	30.48	43.30	9.42 × 10^−6^
Z30: Contraceptive management	10.76	4.42	4.60 × 10^−5^
Z09: Encounter for follow-up examination after completed treatment for conditions other than malignant neoplasm	0.80	4.57	8.40 × 10^−5^
N70: Salpingitis and oophoritis	1.99	7.07	1.16 × 10^−4^
N97: Female infertility	12.75	6.19	1.46 × 10^−4^
N83: Noninflammatory disorders of ovary, fallopian tube and broad ligament	27.49	38.00	2.00 × 10^−4^
N94: Pain and other conditions associated with female genital organs and menstrual cycle	23.11	14.87	4.07 × 10^−4^

Individuals with both endometriosis lesion sites were excluded. Significance is declared at a false discovery rate of 5%

### Endometriosis GWAS

A total of 21 genomic loci reached genome-wide significance (*P* < 5 × 10^−8^), including 19 previously reported risk loci (Rahmioglu et al. 2023; Sapkota et al. 2017) (Supplementary Table 4). Neither of the two novel loci (2:208704347:C:T and 12:115206320:A:G) are within 500 kb of a previously reported endometriosis risk locus. Except for rs3757070, the genome-wide significant loci in the FinnGen GWAS retained genome-wide significance in the GWAS meta-analysis. The estimated genomic inflation factor (*λgc* = 1.06) suggested there was little evidence of systemic bias and results were consistent with an increase in power from the Sapkota et al. (2017) endometriosis meta-analysis. Summary statistics for 7,295,491 SNPs were used in subsequent analyses.

### Genetic correlation between endometriosis and comorbidities

**Fig. 1:**
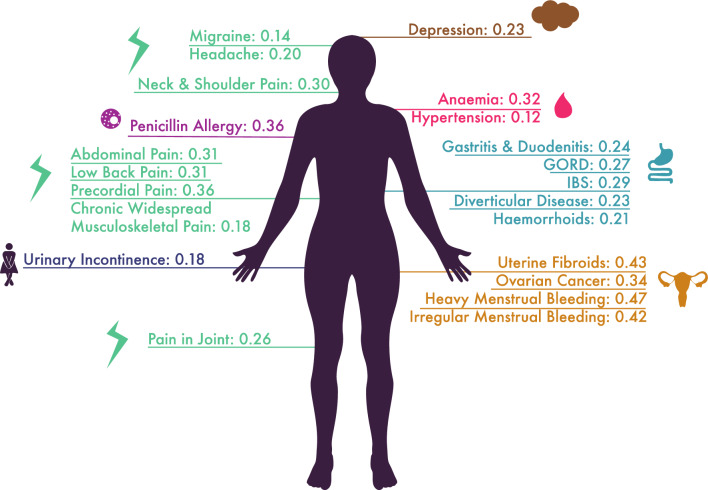
Genetic correlations with endometriosis. The genetic correlation (*r*_g_) of each trait with endometriosis is listed after each trait. Genetic correlations were estimated with LDSC using GWAS summary statistics. Only significant results at an FDR of 5% are illustrated. *GORD* gastroesophageal reflux disease, *IBS* irritable bowel syndrome

The genetic correlation with endometriosis was assessed for 74 traits identified in the UK comorbidity search. Genetic correlation assesses the overall concordance of direction and magnitude of effect of variants across the genome on the two traits. All 74 traits assessed for genetic correlation with endometriosis showed positive epidemiological correlation in the UKB. Of the 74 traits, 22 traits had a significant positive genetic correlation with endometriosis (FDR 5%) (Fig. 1, Supplementary Table 5). Multiple pain, blood-related and gastrointestinal traits showed a significant genetic correlation.

### Shared genetic risk loci between endometriosis and comorbidities

The genetic correlation analysis revealed a genome-wide correlation in genetic effects for 22 traits, however it is not clear whether these correlations are driven by shared genetic variants or nearby distinct variants. Colocalisation analysis, using GWAS-PW, was performed for the 22 traits identified as significantly genetically correlated with endometriosis to identify loci likely harbouring one pleiotropic variant associated with both traits (assessed by PPA3) and loci likely harbouring two causal variants giving rise to each trait independently (assessed by PPA4). Loci with a high confidence (> 90% probability) and suggestive confidence (> 50% probability) of both hypotheses are reported (Table 2, Supplementary Tables 6 and 7). Uterine fibroids had the largest number of shared genetic variants with endometriosis (*n* = 11 for > = 90% probability), whilst hypertension and haemorrhoids had the greatest numbers of loci with two distinct genetic variants (*n* = 12 and *n* = 7, respectively, for > = 90% probability). For all identified loci with PPA3 > = 0.9 (*n* = 30), 13 contained an endometriosis lead variant. Four endometriosis lead variants are within regions shared by endometriosis and two or more other traits. These variants include rs12037376, an intronic variant in *WNT4* which was in an endometriosis risk locus associated with both uterine fibroids and urinary incontinence; rs6546324, an intronic variant in LOC105374786 also associated with gastro-oesophageal reflux disease (GORD) and haemorrhoids; rs6025, a missense variant in *F5*, which was in an endometriosis risk locus associated with diverticular disease and haemorrhoids (and heavy menstrual bleeding with PPA3 = 0.87); and rs635634, an intergenic variant on chromosome 9 which was in an endometriosis risk locus associated with hypertension and ovarian cancer.

**Table 2:** Number of regions for comorbid conditions of endometriosis with strong (> = 0.9) and suggestive (> = 0.5) evidence of having a shared causal variant (PPA3) or two distinct causal variants (PPA4) with endometriosis

Trait	PPA3 > = 0.9	PPA3 > = 0.5	PPA4 > = 0.9	PPA4 > = 0.5
Migraine	0	0	0	1
Headache	3	9	0	1
Neck and shoulder pain	0	0	0	0
Abdominal pain	0	2	0	0
Low back pain	0	0	0	0
Precordial pain	0	0	0	0
Chronic widespread musculoskeletal pain	0	0	0	0
Pain in joint	0	0	0	0
Gastritis/duodenitis	0	0	0	0
Gastro-oesophageal reflux disease	2	8	0	2
Irritable bowel syndrome	1	1	0	0
Diverticular disease	2	4	3	4
Haemorrhoids	4	13	7	24
Uterine fibroids	11	24	2	5
Ovarian cancer	4	7	1	3
Heavy menstrual bleeding	1	2	0	0
Irregular menstrual bleeding	0	0	0	0
Anaemia	0	0	0	0
Hypertension	1	6	12	29
Urinary incontinence	1	1	0	1
Depression	0	5	0	2
Penicillin allergy	0	0	0	0

Analysis was performed in GWAS-PW

### Gene based association analysis of endometriosis and its comorbidities

A gene-based test was conducted to identify potential target genes shared between endometriosis and its comorbidities. fastBAT performed a gene-based association analysis to determine the genes associated with each trait (Bakshi et al. 2016). Gene-based analysis was conducted for the 22 traits identified as significantly genetically correlated with endometriosis. There were fifty genes significantly associated with endometriosis at a genome wide significance threshold (*P* < 0.05/gene total), of which 26 were also significant for at least one other trait (Supplementary Table 8). Uterine fibroids had the greatest number of overlapping genes (*n* = 14) with endometriosis at the genome-wide significance threshold. When considering the top 1% of genes associated with each trait (*n* = 239 for endometriosis), 114 endometriosis-associated genes overlapped with at least one trait (Supplementary Table 9). The genes *MON1A* and *MST1R* were associated with the greatest number of traits: abdominal pain, chronic widespread musculoskeletal pain, GORD, heavy menstrual bleeding and pain in joint. The greatest number of overlapping genes in the top 1% was seen for uterine fibroids (*n* = 26), heavy menstrual bleeding (*n* = 24), GORD (*n* = 21) and ovarian cancer (*n* = 20). Multiple HOXC genes: *HOXC-AS1-3, HOXC4, HOXC5, HOCX6, HOXC8, HOXC9 and HOXC10* were also shared between subsets of GORD, hypertension and precordial pain. The *F5* gene, which was identified to likely harbour a pleiotropic variant between heavy menstrual bleeding, haemorrhoids, diverticular disease and endometriosis in the GWAS-PW analysis, was also identified in the top 1% of genes for these traits. Likewise, *WNT4,* identified to likely harbour a pleiotropic variant between uterine fibroids, urinary incontinence and endometriosis, was identified in the top 1% of genes for these three traits in addition to neck/shoulder pain.

### Causal relationship between endometriosis and comorbidities

To ascertain whether the comorbidity of endometriosis with other traits in the UKB could be explained by causality, MR was applied. Analyses were limited to traits with at least three independent genome-wide significant SNPs, meaning 76 traits were assessed as an outcome of endometriosis, and 41 traits as a cause of endometriosis were assessed (Supplementary Tables 10 and 11). Four MR methods were initially applied to assess each possible relationship: IVW, weighted median, MR-Egger and GSMR. The instrument strength for IVW, weighted median and MR-Egger methods were not subject to weak instrument bias as indicated by the *F*-statistic (endometriosis: *n* = 23, *F* = 57.19, comorbidities: Supplementary Table 11). GSMR could not be conducted for all traits as it has the additional requirement of the effect allele frequency, which is not routinely reported in GWAS summary statistics. Using a stringent Bonferroni multiple testing corrected threshold, *P* < 1.22 × 10^−3^ for endometriosis as outcome, *P* < 6.58 × 10^−4^ for endometriosis as exposure, there were six traits with at least one of IVW, weighted median, MR-Egger and GSMR passing the relevant multiple testing corrected *P* value threshold: ovarian cancer, heavy menstrual bleeding, obesity traits, uterine fibroids, allergic rhinitis and ischaemic heart disease.

A clear causal effect could be ascertained for the genetic liability of endometriosis on ovarian cancer. This is supported by the IVW (beta = 0.20, *P* = 2.59 × 10^−5^), weighted median (beta = 0.18, *P* = 4.67 × 10^−5^) and GSMR (beta = 0.18, *P* = 4.19 × 10^−11^) methods. The MR-Egger estimate was concordant in magnitude and directionality (beta = 0.17), although not significant. Cochran’s *Q* and Rücker’s *Q*′ indicated significant heterogeneity (*P* = 4.59 × 10^−5^, *P* = 2.76 × 10^−5^, respectively), whilst the MR-Egger intercept did not indicate directional pleiotropy. All SNPs passed leave-one out analysis for weighted median and IVW methods. Both IVW (beta = 0.20, *P* = 2.59 × 10^−5^) and weighted median (beta = 0.18, *P* = 2.00 × 10^−5^) methods remained significant upon removal of two SNPs failing MR Steiger filtering and the MR Egger intercept remained non-significant, although heterogeneity remained (Cochran’s *Q* statistic *P* = 4.59 × 10^−5^, Rücker’s *Q*′ statistic *P* = 2.76 × 10^−5^). The MR-PRESSO result agreed with a causative relationship before and after outlier removal (raw: beta = 0.20, *P* = 3.60 × 10^−4^; outlier corrected: beta = 0.18, *P* = 1.35 × 10^−4^). When ovarian cancer was assessed as the exposure variable, no method passed multiple testing correction (Supplementary Table 11).

Initial estimates suggested a causal effect of endometriosis on heavy menstrual bleeding via the MR-Egger (beta = 0.039, *P* = 3.41 × 10^−4^) and GSMR (beta = 0.0079, *P* = 5.53 × 10^−7^) methods. The IVW and weighted median methods had concordant directions of effect and were nominally significant (*P* < 0.05). There was evidence for heterogeneity (Rücker’s *Q*′ statistic *P* = 3.85 × 10^−5^), whilst the MR-Egger intercept had nominal significance (*P* = 3.70 × 10^−3^). Exclusion of the four variants failing leave-one-out MR-Egger analysis rendered it non-significant (*P* = 0.317), with nominally significant heterogeneity (Rücker’s *Q*′ statistic *P* = 0.0293) and non-significant pleiotropy (MR Egger horizontal pleiotropy *P* = 0.625). MR Steiger analysis did not implicate any SNPs as showing greater association with heavy menstrual bleeding than endometriosis. MR-PRESSO identified two SNPs as pleiotropic in the outlier test, resulting in an outlier-corrected estimate (beta = 0.0065, *P* = 0.0121) that significantly differed from the raw estimate (beta = 0.010, *P* = 2.87 × 10^−3^), but did not pass multiple testing correction. No method passed significance with endometriosis as an outcome of heavy menstrual bleeding, however only three independent genome-wide significant SNPs were available for this analysis.

A bidirectional relationship was seen between uterine fibroids and endometriosis, although there was evidence of heterogeneity in effects in both directions. The GWAS participants for uterine fibroids and endometriosis are not independent, which is known to confound the MR result (Hemani et al. 2018). Further investigation of this causal relationship was previously published using the same GWAS summary statistics, bar the overlapping cohorts (Gallagher et al. 2019).

Obesity was identified as enriched in endometriosis cases in the UKB, for which three weight-related female-specific GWAS results were utilised to assess this relationship: WHR, BMI, and WHRadjBMI. BMI did not show significance by any of the four MR methods as a cause or consequence of endometriosis. Increased WHR and WHRadjBMI both passed multiple testing via the GSMR method as an outcome of endometriosis (WHR: beta = 0.025, *P* = 1.13 × 10^−5^; WHRadjBMI: beta = 0.024, *P* = 1.67 × 10^−5^). Increased WHR was also significant for the weighted median method, however, leave one out sensitivity testing indicated that this relationship may be driven by multiple SNPs (*P* > 6.58 × 10^−4^). Upon exclusion of one SNP showing stronger association with WHR phenotypes than endometriosis (MR Steiger test) from the weighted median analysis, the effect for WHR remained significant (WM beta = 0.031, *P* = 2.98 × 10^−4^). MR-PRESSO did not indicate a causative relationship for endometriosis on WHR or WHRadjBMI (WHR raw: beta = 0.013, *P* = 0.427; WHR outlier adjusted: beta = 0.026, *P* = 1.0 × 10^−3^, WHRadjBMI: raw beta = 0.010, *P* = 0.570, WHRadjBMI: outlier adjusted beta = 0.020, *P* = 0.046), in agreement with the MR-Egger and IVW methods. When obesity traits were considered as the exposure variable, GSMR was significant for WHR (beta = 0.13, *P* = 3.38 × 10^−4^) and WHRadjBMI (beta = 0.16, *P* = 1.22 × 10^−7^), whilst MR-Egger, IVW, weighted median and MR-PRESSO were directionally consistent but non-significant. Many obesity-associated SNPs were identified as potentially pleiotropic by GSMR HEIDI analysis, whilst all SNPs passed MR-Steiger testing.

Two traits had significant risk-decreasing effect estimates identified by MR, despite a positive epidemiological correlation in the UKB. A preventative effect of variants associated with allergic rhinitis on endometriosis was identified by the GSMR method (beta = − 2.21, *P* = 6.63 × 10^−4^). The direction of effect was concordant with IVW, weighted median and MR-PRESSO estimates, but not for the MR Egger estimate. All SNPs passed MR-Steiger testing. There was no evidence for endometriosis as a cause of allergic rhinitis. Additionally, a preventative effect of variants associated with ischaemic heart disease on endometriosis was identified via the IVW (beta = − 0.15, *P* = 1.68 × 10^−4^), weighted median (beta = − 0.18, *P* = 1.04 × 10^−3^), and GSMR (beta = − 0.11, *P* = 2.41 × 10^−4^) methods. MR Egger’s intercept provided no evidence for horizonal pleiotropy, however there was nominal significance for heterogeneity estimates. Removal of rs1333042 from IVW reduced the effect’s significance to nominal significance (beta = − 0.093, *P* = 0.012) and eliminated heterogeneity (Cochran’s *Q* statistic *P* = 0.25), while its exclusion from the weighted median method rendered the estimate non-significant (*P* = 0.122). Multiple other SNPs were also found to reduce the significance of the weighted median result below the multiple testing corrected threshold in leave one out analysis. All SNPs passed MR-Steiger testing. There was no significant difference between the raw and outlier-corrected analyses for MR-PRESSO, although only the raw estimate passed multiple-testing correction (beta = − 0.15, *P* = 6.3 × 10^−4^), whilst the outlier-corrected did not (beta = − 0.11, *P* = 2.8 × 10^−3^). The reverse model, the genetic liability to endometriosis causing ischaemic heart disease, was not significant by any method.

## Discussion

The characterisation of the comorbidity of two traits has been facilitated by the availability of large genetic datasets and the development of tools that make inferences about the biological overlap without requiring individual-level data or datasets of individuals with both traits. In this study, analysis of GWAS summary statistics was able to characterise the comorbid nature of endometriosis with other conditions. There were 22 traits with a significant genetic correlation with endometriosis, many of which were novel findings. This analysis highlighted a consistent and strong genome-wide crossover in effects between endometriosis and pain, gastrointestinal and blood-related traits. Interpretation of novel causal inferences with MR was challenged by the presence of heterogeneity and discordant sensitivity tests. Identification of possible pleiotropic variants through colocalisation analysis and shared genes with gene-based analysis provided evidence for pleiotropy as an explanation for comorbidity.

Using the UKB, we identified 292 ICD10 codes associated with an endometriosis diagnosis. Some of these traits have been previously identified as comorbid with endometriosis in epidemiological studies, including uterine fibroids (Gallagher et al. 2019), infertility (Mahmood and Templeton 1991; Meuleman et al. 2009; Petre et al. 2021), menstrual disturbances (Missmer et al. 2004; Nnoaham et al. 2012; Treloar et al. 2010; Wei et al. 2016), multiple pain phenotypes (Signorile et al. 2022), asthma (Peng et al. 2017), irritable bowel syndrome (Chiaffarino et al. 2021), various allergies (Shafrir et al. 2021; Yoshii et al. 2021), depression, anxiety (Chen et al. 2016; Estes et al. 2021; Koller et al. 2023), ischaemic heart disease, hypertension (Okoth et al. 2021), anaemia (Choi et al. 2017), migraine (Yang et al. 2012), and ovarian cancer (Capmas et al. 2021; Saavalainen et al. 2018). Whilst many of the identified ICD10 codes relate to traits previously characterised as comorbid with endometriosis, many novel traits were also identified. Interestingly, there were multiple traits related to shoulder pain: shoulder impingement syndrome, rotator cuff syndrome and adhesive capsulitis of shoulder. Diaphragmatic/thoracic endometriosis has been linked to shoulder pain (Ceccaroni et al. 2013; Nezhat et al. 2014, 2019), although is considered to be a rare condition, affecting 1.5% of individuals with concurrent pelvic endometriosis (Ceccaroni et al. 2013). Diaphragmatic endometriosis has also been linked to pneumothorax (Ceccaroni et al. 2013), which was also associated with endometriosis in the UKB. It is possible that diaphragmatic/thoracic endometriosis-associated shoulder pain may alter shoulder movement, leading to these conditions. Alternatively, their comorbidity could arise due to a confounding factor, ascertainment bias, or misdiagnosis given endometriosis is an unlikely suspect for shoulder pain. Another notable set of traits were those related to infection of female reproductive organs: salpingitis and oophoritis (N70.1, N70.9) and acute vaginitis (N76.0), as well as their inflammation: pelvic inflammatory disease (N73.9), inflammatory disease of uterus (N71.0, N71.9), and inflammatory disease of cervix uteri (N72). Endometriosis development has been hypothesised to be initiated by an innate immune response stimulated by an infection (Khan et al. 2018; Kobayashi et al. 2014; Koninckx et al. 2019). There are some limitations in interpreting the epidemiologically associated traits. Interaction with healthcare services increases the opportunity to be diagnosed with any condition, irrespective of whether the motivation for seeking healthcare was related to that condition. Given most endometriosis patients will undergo multiple interactions with healthcare services to achieve their endometriosis diagnosis, evidenced through the ICD10 chapter 21 (Factors influencing health status and contact with health services) being the second most common chapter in the UKB epidemiological analysis, they have an increased chance of being diagnosed with conditions that may have otherwise been undetected, which may amplify correlations between conditions. In particular, the gynaecological traits may be especially subject to ascertainment bias, as most individuals with endometriosis will have consulted a gynaecologist. This may explain the enrichment of polycystic ovarian syndrome (PCOS) cases in endometriosis comorbidity of PCOS, contradicting claims that they are diametric diseases (Dinsdale et al. 2021) and show inverse comorbidity across populations (Crespi 2021). In fact, 12 of the 34 individuals with both PCOS and endometriosis in the UKB had the same date of diagnosis for endometriosis and PCOS, and 17 had PCOS diagnosed within the same 365-day period. Nevertheless, the relationship remained upon exclusion of these 17 individuals (OR = 2.36, *P* = 1.22 × 10^−3^).

Whilst the UKB offers advantages of its cohort size and comprehensive phenotype data there are limitations to the interpretation of comorbidities of endometriosis in this cohort. The mean age of endometriosis diagnosis in the UKB cohort used in this study was 39.2 years, which substantially differs from recent figures: the mean age of diagnosis reported in a surgically-diagnosed Australian cohort was 26.7 years (O'Hara et al. 2020), and a self-report Canadian cohort reported 27.9 years (Singh et al. 2020). Further, we used an age-matched cohort as unrelated endometriosis cases were typically younger than endometriosis-free individuals in the UKB. This disparity likely owes to the dated nature of the cohort: UKB participants included in this study were born between 1937 and 1970, meaning their endometriosis diagnoses occurred when there was less awareness of endometriosis, which may translate to longer diagnostic delays and lower diagnosis rates. Accordingly, in a study published in 1996 of 134 women in the UK the mean age at diagnosis was 32.6 years (Hadfield et al. 1996). Given the difficulties in attaining an endometriosis diagnosis, age of diagnosis is an inappropriate proxy for the age of disease onset, both in the UK Biobank and more recent cohorts. Also, the frequency of endometriosis diagnosis in the UKB is substantially less than the current population frequency, suggesting many diagnoses are missed (Blass et al. 2022). Consequently, it is unclear from the UK Biobank epidemiological data whether the comorbidities identified are useful for prediction of endometriosis. The conditions diagnosed on average before endometriosis are generally restricted to pregnancy. Nevertheless, a recently published study found irritable bowel syndrome and menstrual cycle length as useful predictors of endometriosis in the UKB (Blass et al. 2022). Therefore, we recommend the replication of this comorbidity search and investigation of their predictive capacity in other large health registries. Regardless of these limitations, the validation of many of these traits as biologically overlapping with endometriosis using genetic data suggests the UKB is a valuable resource to explore shared aetiological and pathogenic pathways between diseases.

Several of the traits genetically correlated with endometriosis can be grouped into distinct biological categories. These include pain related traits, menstrual and blood related traits, and gastrointestinal-related traits. Meanwhile, ovarian cancer was the only cancer with a significant genetic correlation; depression, but not anxiety was genetically correlated; and the only inflammatory trait that reached significance was penicillin allergy. This may highlight the importance of disease-specific features as overlapping with endometriosis, rather than features shared between traits within a biological category. However, there were few significant genetic correlation results for which the GWAS summary statistics for the comorbid trait was generated from less than 5000 cases, suggesting the genetic correlation with endometriosis should be re-estimated upon the generation of better-powered GWAS summary statistics.

Pain is a defining feature of endometriosis. Multiple pain traits: abdominal, low back, joint, neck and shoulder, precordial, chronic widespread musculoskeletal pain, as well as headache and migraine, all showed significant genetic correlation with endometriosis. A significant genetic correlation of endometriosis with pain in multiple bodily locations has previously been reported (Rahmioglu et al. 2023). A significant genetic correlation of migraine with endometriosis in this study (*r*_g_ = 0.14) is supported by two studies: Adewuyi et al. (2020) identified a genetic correlation of 0.14 using similar endometriosis summary statistics and independent, greater powered migraine summary statistics, and Rahmioglu et al. (2023) identified a significant genetic correlation of 0.29 using GWAS summary statistics of greater sample size for both endometriosis and migraine. Further, both our study and Adewuyi et al. (2020) present no evidence of a causal relationship. In addition to migraine, none of these pain traits could be linked to endometriosis via causality in the MR analysis. Disease suspicion due to pelvic pain and/or infertility does not guarantee endometriosis will be detected upon laparoscopy (Fernando et al. 2013; Kazanegra et al. 2008; Stegmann et al. 2008). Further, pain correlates poorly with endometriosis severity (Vercellini et al. 2007), and surgical excision of the endometriosis lesions is not always sufficient to alleviate pain (Abbott et al. 2004; Sutton et al. 1994; Vercellini et al. 2006). Therefore, whilst pain is strongly associated with endometriosis, its presence is likely not solely responsible for pain (Maddern et al. 2020). The absence of a causal effect but concordant genome-wide genetic effects supports this interpretation.

Whilst endometriosis has been epidemiologically linked to multiple cancers, the strongest evidence is for an association with ovarian cancer. Here, we assessed the crossover between endometriosis and melanoma, breast, endometrial and epithelial ovarian cancer. A significant genetic correlation and a causal effect of endometriosis on epithelial ovarian cancer (Mortlock et al. 2022; Rueda-Martinez et al. 2021; Yarmolinsky et al. 2019) has been previously published and was supported in our study. We examined epithelial ovarian cancer broadly, whilst histotype-specific analyses have indicated causality and genetic correlation with only a subset of the histotypes (primarily clear cell and endometroid) (Mortlock et al. 2022; Rueda-Martinez et al. 2021; Yarmolinsky et al. 2019). We did not find a genetic correlation or a causal relationship between endometriosis and endometrial cancer, supporting Kho et al. (2021) and Rueda-Martinez et al. (2021). Painter et al. (2018) has published a significant genetic correlation of endometrial cancer with endometriosis, however the datasets used in the present study and by Kho et al. (2021) have increased power. Yang et al. (2021) reports a significant genetic correlation of endometriosis with melanoma in females (*r*_g_ = 0.144, *P* = 0.025), whilst in this study we estimate a very similar correlation coefficient the relationship was not statistically significant (*r*_g_ = 0.134, *P* = 0.078). Similarly, Yang et al. (2021) found a small causative effect of melanoma on endometriosis (beta_GSMR_ = 0.05, *P*_GSMR_ = 0.01) that replicated in this study (beta_GSMR_ = 0.04, *P*_GSMR_ = 0.037), however, using a more stringent multiple testing correction threshold this was not considered statistically significant. Further, the MR-Egger and weighted-median methods were not directionally consistent with the GSMR and IVW results. Therefore, the link between melanoma and endometriosis should be considered cautiously. We also find no evidence of endometriosis causing breast cancer, supporting Rueda-Martinez et al. (2021). Therefore, evidence for a genetic association between endometriosis and malignancies seems to be limited to ovarian cancer.

Depression and anxiety have been previously implicated as genetically correlated with, and as a cause of, endometriosis (Adewuyi et al. 2021; Koller et al. 2023). Here, we did not identify a significant genetic correlation with anxiety, however this may be because the anxiety summary statistics were not female specific, whilst Koller et al. (2023) used female-specific anxiety summary statistics. For depression, we report a significant genetic correlation with endometriosis (*r*_g_ = 0.23, *P* = 8.84 × 10^−9^), supporting previously published results [*r*_g_ = 0.34, *P* = 1 × 10^−5^ (Koller et al. 2023), *r*_g_ = 0.27, *P* = 8.85 × 10^−27^ (Adewuyi et al. 2021)]. In this study the effect of depression on endometriosis was not considered significant (beta_GSMR_ = 0.18, *P*_GSMR_ = 0.006) due to our highly stringent multiple testing burden. Our study does not replicate the Adewuyi et al. (2021) significant IVW and weighted-median test at *P* < 0.05, although the effect sizes were similar, which is expected given the same depression GWAS data and similar endometriosis GWAS data was used. Using an alternative approach of one-sample MR in the UK Biobank, Koller et al. (2023) identified a small causative effect (OR = 1.09, *P* = 2 × 10^−16^) of depression on endometriosis. Koller et al. (2023) further investigated whether inflammatory pathways were involved in the causative effect of psychiatric traits on endometriosis by testing the effect of C-reactive protein on endometriosis. No causative effect was identified, although the C-reactive protein GWAS data was not female-specific (Koller et al. 2023). This suggests that a causal mechanism of depression on endometriosis requires further investigation.

Given endometriosis is an inflammatory condition, an overlap with other inflammatory traits may be anticipated. Yet, we did not find a causal relationship with any inflammatory condition, and the only immune-related trait with a significant genetic correlation was penicillin allergy. A significant genetic correlation of endometriosis with osteoarthritis has been previously reported but was not investigated here (Rahmioglu et al. 2023). A significant genetic correlation with asthma has been previously reported [*r*_g_ = 0.155, *P* = 0.033 (Adewuyi et al. 2022) and *r*_g_ = 0.178,* P* = 5.59 × 10^−4^ (Rahmioglu et al. 2023)], however it was not supported by this study (*r*_g_ = 0.0796, *P* = 0.057). Similar GWAS datasets for endometriosis and asthma were used between our study and Adewuyi et al. (2022), whilst Rahmioglu et al. (2023) used independent asthma GWAS data generated from fewer cases and overlapping, greater powered endometriosis GWAS data. An absence of a genetic correlation for systemic lupus erythematosus, rheumatoid arthritis, ulcerative colitis and Crohn’s disease has previously been reported, supporting the results of this study (Rahmioglu et al. 2023). We also replicated an absence of a causal relationship between asthma and endometriosis (Adewuyi et al. 2022), and for systemic lupus erythematosus and rheumatoid arthritis on endometriosis (Garitazelaia et al. 2021).

A significant genetic correlation between endometriosis and gastritis/duodenitis, as well as GORD, has been previously reported (Adewuyi et al. 2021) and was replicated in this study. We further found a significant genetic correlation with other gastric traits: diverticular disease, irritable bowel syndrome and haemorrhoids, further consolidating the comorbidity between endometriosis and gastric traits. Adewuyi et al. (2021) also reported a causal effect of GORD on endometriosis by the IVW method in the absence of heterogeneity or pleiotropy, supported by MR-PRESSO (not supported by MR-Egger or weighted median), which we did not replicate with IVW, despite using better powered endometriosis summary statistics. Our results are concordant in a lack of causation of endometriosis on gastritis/duodenitis and GORD.

The relationship between endometriosis and body weight has been contentiously debated owing to inconsistent findings by epidemiological studies. Here we saw an increased risk of both obesity and abnormal weight loss in individuals with endometriosis, and anorexia in surgically confirmed endometriosis cases in the UKB. Previous reports suggest a higher BMI may be associated with a decreased risk for endometriosis (Liu and Zhang 2017), whereas another study reports no effect of being overweight or obese on endometriosis, conversely being underweight was a risk factor (Jenabi et al. 2019). Women with BMIs in the obese range have increased disease severity compared to women with normal and pre-obese BMIs (Holdsworth-Carson et al. 2018). We did not find a significant genetic correlation between endometriosis and BMI, WHR or WHRadjBMI in females. This supports previous findings that while several risk loci are likely shared between endometriosis and weight traits, they do not have a concordant genome-wide directional effects (Rahmioglu et al. 2015). However, a genetic correlation with female BMI (*r*_g_ = 0.12, *P* = 3.45 × 10^−7^), but not WHRadjBMI has been reported using better powered endometriosis summary statistics and the same anthropometric summary statistics (Rahmioglu et al. 2023). Garitazelaia et al. (2021) found a preventative effect of increased weight and BMI on endometriosis, but this was not replicated in sensitivity tests, and pleiotropic effects of the instrumental variables were not investigated. Further, they did not find an effect of WHR on endometriosis (Garitazelaia et al. 2021). Venkatesh et al. (2022) found a causal effect of increased WHR and WHRadjBMI on endometriosis was mediated by leptin and insulin, whilst there was no evidence for a causal effect of endometriosis on WHR, WHRadjBMI or BMI (Venkatesh et al. 2022). Here, we did not find robust evidence for a causal relationship between WHR, WHRadjBMI or BMI and endometriosis. The GSMR method implicated a bidirectional causal relationship for WHR and WHRadjBMI with endometriosis, although the effect estimates across methods were greater for endometriosis as the outcome variable. However, this could be attributed to the increased power (sample size) in the WHR and WHRadjBMI GWASs. Endometriosis likely onsets in adolescence in most cases, so the use of adult-associated obesity SNPs to assess the causality of obesity on endometriosis may not be appropriate. Childhood BMI (male and female combined) has been assessed as a cause of endometriosis using MR, for which a clear causative effect could not be discerned (Yan et al. 2022). Permitting the availability of such data, we recommend the assessment of endometriosis as an outcome of body weight using female-specific, adolescence associated SNPs.

The relationship between heavy menstrual bleeding and endometriosis was of particular interest given the increased prevalence of heavy menstrual bleeding in endometriosis cases has been used to support Sampson’s theory of retrograde menstruation through increased exposure to menstruation. Unfortunately, the heavy menstrual bleeding summary statistics were not well powered, with only three genome-wide significant SNPs, which impeded consideration of heavy menstrual bleeding as a cause of endometriosis. Heavy menstrual bleeding was deemed unlikely an outcome of endometriosis given the relationship was not robust to the removal of several SNPs. Two SNPs, rs6025 and rs11031005 identified as outliers in MR were also identified by GWAS-PW as likely causal of both traits. rs6025 is a coding variant in *F5* causing increased blood clotting and is a risk-decreasing SNP for endometriosis and heavy menstrual bleeding. rs11031005 is in the *FSHB* locus, which contains several variants in a haplotype associated with multiple other female reproductive traits (McGrath et al. 2021). Therefore, the relationship between endometriosis and heavy menstrual may be driven by pleiotropy, however better-powered heavy menstrual bleeding data is necessary to comprehensively assess whether a causal effect of heavy menstrual bleeding on endometriosis also plays a role. A menstrual abnormality, such as heavy menstrual bleeding, is a common motivating factor for consulting a gynaecologist. Therefore, it is suspected endometriosis cohorts will be enriched for these motivating factors, irrespective of whether these factors are biologically linked to the diagnosed disease. Disentangling whether heavy menstrual bleeding is truly a feature of endometriosis will be of interest in future studies, where increased-powered heavy menstrual bleeding GWAS data is available.

The *F5* gene was identified as likely harbouring a pleiotropic variant (rs6025) for endometriosis, diverticular disease, heavy menstrual bleeding and haemorrhoids. The gene was also identified in the gene-based analysis of all these traits except diverticular disease. The *F5* gene encodes a protein involved in the coagulation cascade. The lead endometriosis mutation in the region identified by GWAS-PW is rs6025, a missense variant (p.Arg534Gln), which has a protective effect on endometriosis (beta = − 0.27), heavy menstrual bleeding (beta = − 0.011), diverticular disease (beta = − 0.0077), and haemorrhoids (beta = − 0.11). This variant has been previously characterised as a risk factor for venous thrombosis (Hillarp et al. 1995). The rs6025 mutation occurs at a cleavage site used by activated protein C, thereby slowing down the deactivation of F5, allowing it to continue to promote the generation of the blood clotting factor thrombin (Heeb et al. 1995). As this variant is protective for endometriosis, this suggests that increased clotting may reduce the risk of endometriosis. Indeed, alterations in coagulation factors has been seen in endometriosis cases (Ottolina et al. 2020).

In the gene-based analysis, many genes with roles across multiple traits were identified. The HOX genes, particularly the *HOXC* cluster was a notable result, being identified in the top 1% of genes of endometriosis, GORD, hypertension and precordial pain. The differential expression of the HOX genes controls the development of the female reproductive tract: *HOXA9* controls the development of the fallopian tubes, whilst *HOXA11* controls the development of the cervix (Du and Taylor 2015). The HOX genes remain important in adulthood; *HOXA11* has also been implicated in endometriosis-associated infertility, as it fails to rise in patients with endometriosis during the implantation window and secretory phase (Du and Taylor 2015). There is evidence for differential methylation in ectopic, eutopic and control endometrial tissue for *HOXA11* and *HOXC10*, but not for *HOXC9* (Esfandiari et al. 2021). *HOXC* genes are not well characterised, however, are hypothesised to have a role in endometrial proliferation, as compared to the endometrial differentiation role of *HOXA* genes (Akbas and Taylor 2004). The role of the *HOXC* genes in all these traits requires further investigation.

There are a few limitations to the genetic analyses. For most comorbidities investigated, sex-specific GWAS summary statistics were not available, so data utilised were from combined male–female datasets. Differences in the genetic architecture between males and females is an underexplored area, but is known to be substantial for some traits, e.g., testosterone where there is a null genetic correlation between males and females (Ruth et al. 2020). Sex-specific GWAS summary statistics may be necessary to identify causal relationships with endometriosis and shared genetic risk loci. Likewise, GWAS summary statistics of increased power may be needed to discover such associations. Therefore, non-significant MR results may not be sufficiently powered, so should not be conclusively considered evidence for a lack of causation. Furthermore, low probabilities in the colocalisation analysis may be driven by poorly powered data. We note that the gene-based analysis method assumes the relevant gene is nearby the variant; however, gene-variant regulation is often more complex (Braenne et al. 2015; Sanyal et al. 2012; Zhu et al. 2016), therefore not all identified genes may truly be shared between disorders. Lastly, GWAS-PW may fail to identify two independent causal variants in the presence of very high LD (Pickrell et al. 2016).

We have performed a comprehensive search for comorbidities of endometriosis in the UKB and have characterised the comorbid nature of endometriosis using genomic data. We highlight the heterogeneity of endometriosis through the diverse range of comorbidities experienced by women with the disease. The identification of many traits epidemiologically correlated with endometriosis is informative for future studies, as they represent candidates for disease prediction, but require validation in large, contemporary epidemiological databases. Our analyses suggest that the comorbid relationships of endometriosis is largely due to shared risk variants and genes, rather than causality. These shared genes and risk variants provide insight into the developmental origins of endometriosis.

## Supplementary Information

Below is the link to the electronic supplementary material.

Supplementary file1 (XLSX 228 KB)

## Data Availability

Data can be accessed from UK Biobank (https://www.ukbiobank.ac.uk/) as per their published data access procedures. Summary data for the FinnGen Endometriosis GWAS is available from their results portal (https://www.finngen.fi/en/access_results). Endometriosis GWAS summary statistics are available on request. Other publicly available GWAS summary data are available from the GWAS Catalog (https://www.ebi.ac.uk/gwas/), or as cited in Supplementary Table 1. Any additional data supporting the conclusions of this article are included within the article and its supplementary files.
